# Reducing the rate of central line-associated bloodstream infections; a quality improvement project

**DOI:** 10.1186/s12879-023-08744-5

**Published:** 2023-10-30

**Authors:** David Odada, Hellen Munyi, Japhet Gatuiku, Ruth Thuku, Jared Nyandigisi, Anne Wangui, Emilie Ashihundu, Beatrice Nyakiringa, Jemimah Kimeu, Martin Musumbi, Rodney D. Adam

**Affiliations:** 1 Department of Nursing, Aga Khan University Hospital, Nairobi, Kenya; 2 Department of Pharmacy, Aga Khan University Hospital, Nairobi, Kenya; 3 Department of Quality, Aga Khan University Hospital, Nairobi, Kenya; 4 Department of Internal Medicine, Aga Khan University Hospital, Nairobi, Kenya; 5 Department of Pathology, Aga Khan University Hospital, Nairobi, Kenya

**Keywords:** CLABSI- central line-associated bloodstream Infections, CQI- continuous quality improvement

## Abstract

**Background:**

The burden of central line-associated bloodstream infections is significant and has negative implications for healthcare, increasing morbidity and mortality risks, increasing inpatient hospital stays, and increasing the cost of hospitalization. Efforts to reduce the incidence of central line-associated bloodstream infections have utilized quality improvement projects that implement, measure, and monitor outcomes. However, variations in location, healthcare organization, patient risks, and practice gaps are key to the success of interventions and approaches. This study aims to evaluate interventions of a quality improvement project on the reduction of central line-associated bloodstream infection rates at a university teaching hospital.

**Methods:**

This was a retrospective review of a quality improvement project that was implemented using the Plan-Do-Study-Act quality improvement cycle. Active surveillance of processes and outcomes was performed in the critical care areas; compliance to central line care bundles, and central line-associated bloodstream infections. Interrupted time series was used to analyze trends pre and post-intervention and regression modeling to estimate data segments preceding and succeeding the interventions.

**Results:**

There were 350 central line insertions, 3912 catheter days, and 20 central line-associated bloodstream infection events during the intervention period. Compliance with central line care bundles was at 94%. There was a trend in the reduction of central line-associated bloodstream infections by 18% that did not reach statistical significance (p = 0.252).

**Conclusions:**

Improvement projects to reduce central line-associated bloodstream infections face challenges and complexities associated with implementing interventions in real-world healthcare settings. There is a great need to continuously monitor, evaluate, readjust, and adapt interventions to achieve desired results, sustain improvements in patient outcomes, and investigate reasons for non-adherence as keys to achieving desired outcomes.

## Introduction

Central Line-Associated Bloodstream Infections (CLABSI) are hospital-acquired infections that are common yet preventable. They are associated with an increase in morbidity and mortality, prolonged hospital stay, and increased treatment costs [1]. Practices associated with the occurrence of CLABSI include lack of or inadequate knowledge and training in the care of central venous catheters (CVC), gaps in aseptic technique when inserting and maintaining central venous catheters, and overuse of antibiotics [2, 3]. With increased attention on healthcare-associated infection (HAI) and surveillance being part of infection prevention and control function, healthcare facilities are collecting standardized data on HAIs to track internal performance and also compare these outcomes with national and international benchmarks [4]. Healthcare facilities require a sustained effort to ensure that the occurrence of healthcare-associated infections such as CLABSI is kept at the lowest rates possible.

Estimates from the United States indicate that 30,000 to 40,000 cases of CLABSI occur yearly [5]. Populations most at risk are patients in critical care units because of the extensive use of central venous catheters. In addition, central venous catheters are frequently placed in emergency circumstances, repeatedly accessed daily, and used for extended durations [6]. The magnitude of CLABSI in lower- and middle-income countries is unknown because national surveillance systems on HAIs are either not in place or under-established. This has made inter-hospital comparisons and benchmarking of CLABSI rates difficult because there is lack of uniformity across institutions. Unavailability of regional surveillance systems makes comparing performance with external benchmarks challenging because of disparities in resources, healthcare systems, and patient risks [7]. Another challenge is the potential of reporting infection rates and unrealistic prevention strategies due to the labor-intensive process of monitoring process compliance and CLABSI case finding being executed by infection prevention and control practitioners [8, 9]. Some creative strategies that have been recommended are practical only with an electronic health record system [6, 9].

Successful strategies for reducing CLABSI in healthcare have been demonstrated by formal theory and studies done in different settings. CLABSI prevention efforts vary with different locations or patient populations according to outcome data and risk assessment [10]. While the Centers for Disease Control and Prevention (CDC) has provided evidence-based guidelines for the prevention of CLABSI, it is worth noting that quality improvement approaches have less generalizability outside the setting in which they are implemented [6]. A significant association between central line bundle compliance and CLABSI reduction has been demonstrated in studies done in different settings although variations exist on what elements of the central line bundles are included as interventions [6]. Studies have supported the importance of staff training on central line care and compliance with central line insertion and maintenance bundles in CLABSI prevention [11]. Another important practice is to have a process in place to ensure adherence to infection prevention practices at the time of central venous catheter insertion and maintenance using a checklist [10]. Central line insertion practices included in the bundles are hand hygiene before CVC insertion, use of maximum sterile barrier precautions during CVC insertion, use of alcohol and chlorhexidine solution for skin preparation, and use of chlorhexidine-containing dressings for CVCs in patients over 2 months of age. Measurements of CLABSI incidence rates (number per 1,000 catheter days) and data should also be shared regularly with relevant teams [10, 12–15]. Overall compliance for the central line is also measured in percentage as compliance to “ALL OR NONE’’ of the bundle’s elements and shared [16].

These prevention strategies have expanded, and the experience of the implementation of these strategies has also increased [10]. Increased rates of central line-associated bloodstream infection remain a significant challenge in many healthcare institutions despite the application of evidence-based interventions in practice [6]. It is assumed that healthcare facilities have been unsuccessful because they overlook informal and formal theories in planning and executing improvement efforts [17]. There is also limited scholarly literature on how outcomes vary in healthcare settings if quality improvement project models are used [18]. To produce better local processes and products, models such as Plan Do Study and Act have been widely recommended for quality improvement projects [19], a description of a successful or a failure of strategies after the implementation of interventions would help other healthcare facilities decide what interventions to adopt in practice [20]. Evidence from quality improvement reports shows that quality improvement projects can produce results in practice and can provide ‘proof of concept’ for replication in other settings [21]. A successful outcome of reduced central line-associated bloodstream infection occurrence is expected by applying central line care bundles in practice and active surveillance of central line-associated bloodstream infection through effective use of theory in actual improvement [1, 8, 17, 22].

This study aims to evaluate interventions of a quality improvement project on the reduction of central line-associated bloodstream infection rates at a university teaching hospital.

## Methods

### Context

The hospital has a capacity of 280 beds with 46 adult critical care beds (ICU, HDU, CCU, and CTICU). The hospital laboratory is accredited by the South African National Accreditation System (SANAS) and by the College of American Pathologists [23]. The microbiology laboratory utilizes the Becton Dickinson Bactec system for continuous blood culture monitoring and the bioMerieux Vitek2 for the identification of organisms.

A project team of 11 members was selected to implement the quality improvement project. Nearly all central lines in the hospital are used in the four critical care units that were chosen for the intervention. Interventions identified to reduce the rates of CLABSI were compliance with CVC insertion and maintenance bundles and active surveillance. Compliance rates for CVC insertion and maintenance bundles were initially assessed using a retrospective review of medical records pre-intervention. The interventions for this improvement project transitioned to real-time observation of practices to monitor compliance with bundle items using a standardized bundle checklist to accurately monitor practice, improve compliance and standardize audit process. Positive behavior of compliance to prescribed practice of adherence to CVC care bundles would be influenced through direct observations of practice.

### Interventions

**Fig. 1:**
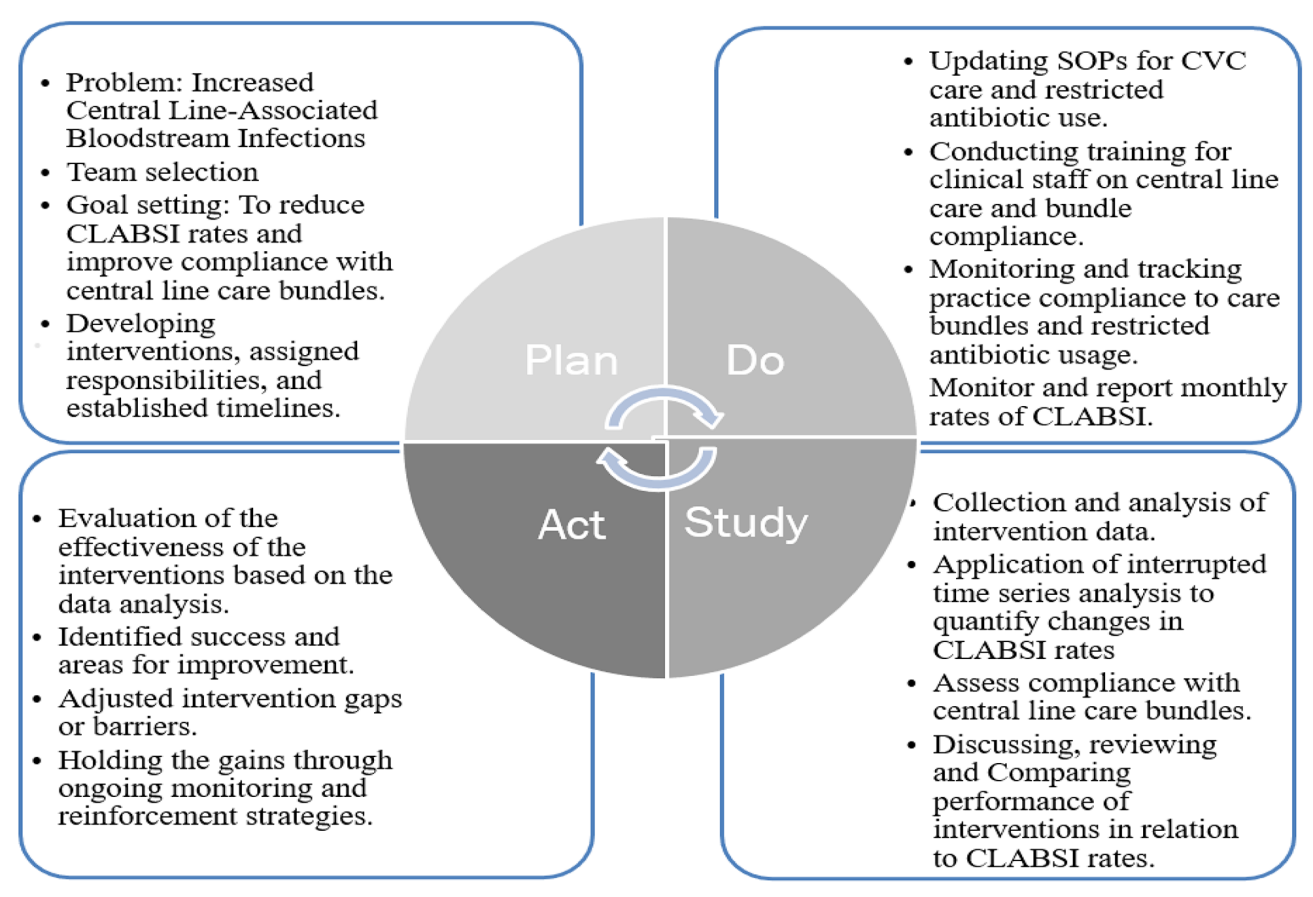
PDSA Implementation Framework

Clinical staff, doctors, and nurses, in the four critical units were engaged to brainstorm on the causes for increased central line-associated bloodstream infections. A Plan, Do, Study, Act (PDSA) model was used as a framework to guide the improvement project (Fig. 1).

During the “Plan” phase, the problem was identified as high rates in central line associated bloodstream infections and inaccurate compliance rates with central venous catheter insertion and maintenance bundles. The objective was to carry out a monthly surveillance and tracking of CLABSI rates and monitor practice compliance to CVC bundles through direct practice observations.

For the “Do” phase, the standard operating procedures for central line care bundles was reviewed, updated and shared with all staff, practice monitoring and tracking checklists for bundles compliance was standardized, staff were retrained on central line care bundles, and utilized the Centers for Disease and National Health and Safety Network (CDC/NSHN) criteria for CLABSI surveillance was reinforced to track cases.

The “study/ check” phase the project team analysed the compliance rates of CVC bundles and in relation to the CLABSI rates, compared bundle compliance data to previous performance to assess improvement, and convened a monthly multidisciplinary team meetings for an update of the team members roles and responsibilities and review and discuss project performance.

In the “Act” phase, we evaluated the effectiveness of bundle compliance in reducing CLABSI rates, identified the successes and areas for improvement of the improvement project, and made necessary adjustments to interventions while maintaining progress.

The National Health and Safety Network (NSHN) defines a central venous catheter (CVC) as an intravascular access device or catheter that terminates at or near the heart or in one of the major vessels, including the pulmonary artery, superior vena cava, inferior vena cava, brachiocephalic veins, internal jugular veins, subclavian veins, external iliac veins, common iliac veins, or femoral veins [24]. Central line insertion bundle components that were actively implemented were compliance with hand hygiene before central line insertion, maximum barrier by staff while performing central line insertion, using a sterile drape on the insertion site, use of alcohol and chlorhexidine antiseptic for skin preparation, and allowing the site to air dry, and use of chlorhexidine-containing dressings. Compliance was calculated by dividing the total number of compliances to all five items of insertion bundles by the total number of observations for the CVC insertion bundle multiplied by 100. Maintenance bundle elements included hand hygiene before manipulating a central line, maintaining a clean and intact CVC, changing intravenous tubing every 72 h, ensuring stopcocks have dead-end sterile caps and are swabbed with a chlorohexidine antiseptic before access, and daily assessments of CVC necessity. Failure to adhere to all the bundle items was considered as “bundles not met” since it increased the risk of contamination of the central line that may result to CLABSI. Direct practice observation of central line insertions and maintenance of bundles was conducted by the unit clinical Nurse educator who were not directly involved in the procedures. Standardized bundle checklist was used during observations of practice compliance. Whenever a central venous catheter (CVC) insertion procedure was scheduled, a clinical nurse educator would directly observe the insertion to establish if all CVC bundle checklist items were met or not met. Central line insertions was based on clinical needs of patients, the number of samples to observed was determined by forecasted insertions from previous months number of insertions. For maintenance bundles, clinical nurse educator using RAND sampling for days and patients with Central lines used a CVC maintenance checklist to observe and assess for compliance to bundle items.

Surveillance for CLABSI was done both manually and electronically since our electronic health record at the time only supported laboratory and radiology. Patients’ notes was still using a manual system. Identifying patients who met the criteria for CLABSI involved extensive manual chart reviews to collect information of CVC insertions, utilization and removal. The infection prevention and control (IPC) team, infectious disease team, and critical care clinical teams collectively reviewed cases and determined if they met the National Health and Safety Network (NHSN) criteria for CLABSI, “lab-confirmed bloodstream infection in a patient who has had a central line for at least 48 hours on the date of the development of the bloodstream infection and without another known source of infection” and having the central line removed not more than 24 h prior to the date of a positive blood culture [12]. CLABSI rates (cases per 1000 catheter days) for each unit were then aggregated and reported monthly.

### Statistical analysis

Interrupted time series (ITS) analysis was used to quantify changes in level and trend from pre- and during-intervention and assess if the estimated differences are statistically significant. Regression modeling was used to estimate coefficients for both the data segment preceding and succeeding an intervention [20]. The fitted regression equation model for the ITS is as follows; Y = β_0_ + β_1_(T)+β_2_(Xt)+β_3_(TXt)+εt Where Y represents the dependent variable.

T: Time elapsed since the beginning of the study.

Xt: Dummy variable representing the pre-and post-intervention period coded 0 and 1 respectively.

TXt: Dummy variable for the time lapsed after intervention.

β_0_: Baseline coefficient at the beginning of the study at T = 0.

β_1_: The change in the dependent variable when there is a unit change in time.

β_2_: The change in the dependent variable during the intervention period.

β_3_: The change in the dependent variable after the intervention period.

εt: Error term.

## Results

There were 260 central line insertions, 3171 central line days, and 28 CLABSI events during the pre-intervention (April 2020 to March 2021), and 350 central line insertions, 3912 central days, and 22 CLABSI events during the intervention (April 2021 to March 2022). Cumulatively the CLABSI rate before the intervention period was 8.83 and 5.62 after the intervention. With regards to compliance to insertion bundles, the average compliance rate during the pre-intervention period was 97%, and in the period after the intervention, the compliance rate increased to 98%, while the compliance rate to maintenance bundles increased from 92 to 98% after the intervention.

### Correlation analysis

A correlation analysis was conducted to assess the nature of the association between compliance with care bundles and CLABSI rates. The pre-intervention period showed that there was a low positive association between compliance with maintenance bundles (r = 0.393) and insertion bundles (r = 0.285) and the CLABSI rate (Table 1). As shown in the table below, these results were not statistically significant.

**Table 1:** Correlations

		Rate	Maintenance Bundles	Insertion Bundles
Rate	Pearson Correlation	1	0.393	0.285
	Sig. (2-tailed)		0.206	0.370
	N	12	12	12
Maintenance Bundles	Pearson Correlation	0.393	1	0.236
	Sig. (2-tailed)	0.206		0.460
	N	12	12	12
Insertion Bundles	Pearson Correlation	0.285	0.236	1
	Sig. (2-tailed)	0.370	0.460	
	N	12	12	12

a. Intervention = Pre-intervention

After the intervention, there was an inverse relationship between compliance with insertion bundles (r = -486, p = 0.109). However, compliance with maintenance bundles still showed a positive association even after the intervention (r = 0.265, p-value 0.404), (Table 2).

**Table 2:** Correlations

		Rate	Maintenance Bundles	Insertion Bundles
Rate	Pearson Correlation	1	0.265	− 0.486
	Sig. (2-tailed)		0.404	0.109
	N	12	12	12
Maintenance Bundles	Pearson Correlation	0.265	1	− 0.264
	Sig. (2-tailed)	0.404		0.407
	N	12	12	12
Insertion Bundles	Pearson Correlation	− 0.486	− 0.264	1
	Sig. (2-tailed)	0.109	0.407	
	N	12	12	12

a. Intervention = After Intervention

**Fig. 2:**
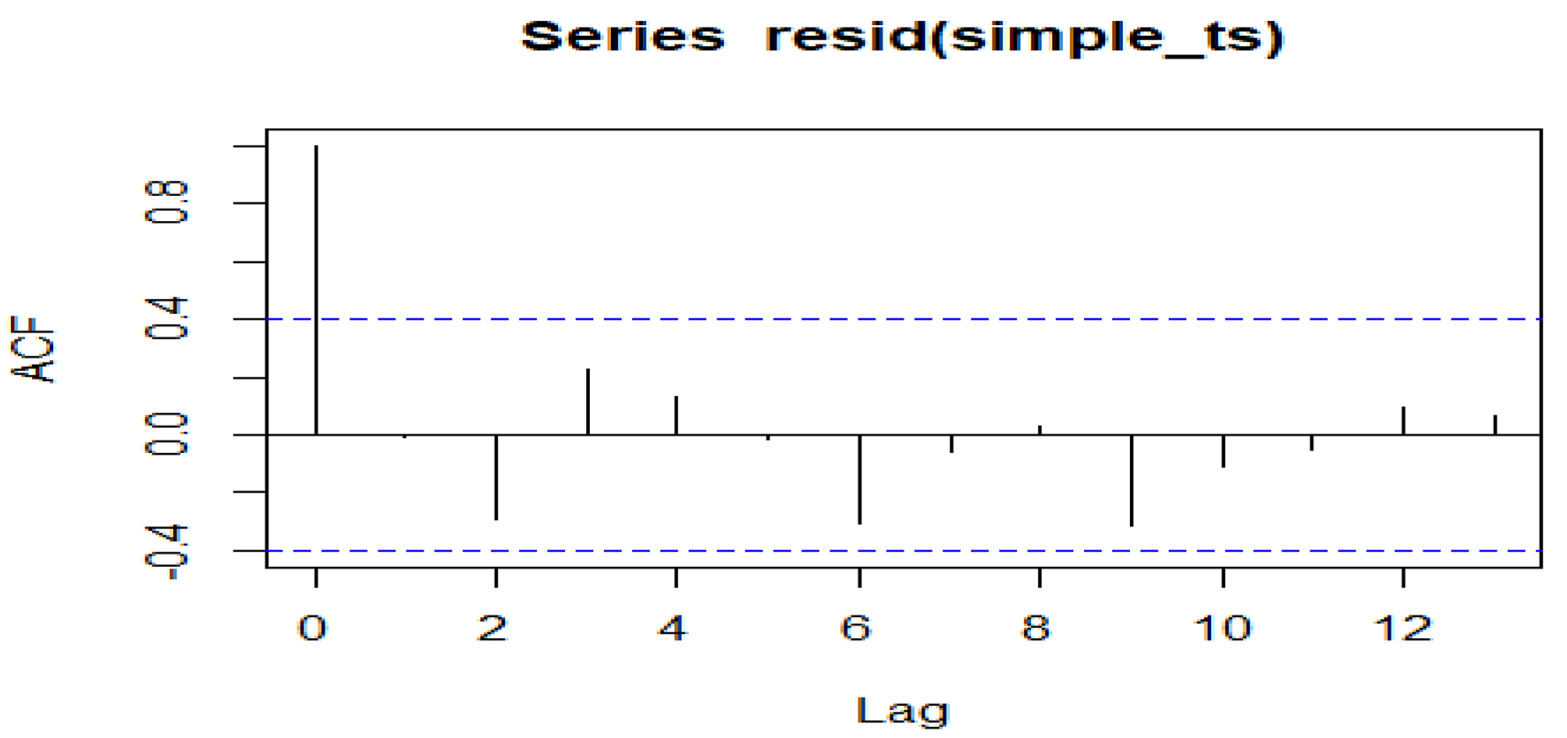
Auto Correlation Function Plot. Durbin-Watson test. data: model1. DW = 2.151, p-value = 0.3814. Alternative hypothesis: true autocorrelation is greater than 0

An interrupted time series analysis was further used to assess if the observed decline in the CLABSI rate following the bundle compliance intervention was statistically significant. Durbin-Watson (DW) test was used to check for autocorrelations in the residuals before running the ITS model and found no evidence of autocorrelation (p = 0.381). Further plotting the autocorrelation function showed all lags after the first lag were within the control limits an indication of no autocorrelation (Fig. 2).

From the segmented regression analysis coefficients, the intercept value of 6.069 represents the estimated CLABSI rate at the beginning of the time series, assuming all other variables were held constant. During the twelve months before the intervention period the CLABSI rate was decreasing by 0.02 every month p-value (0.965), however, there was an immediate estimated increase in the rates during the intervention period by 5.791 (p-value of 0.218), and in the period after the intervention, the rate decreased by 0.947 every month p-value 0.165 (Table 3). These findings were not statistically significant despite the intervention contributing to 18% (Multiple R-squared: 0.181) of the subsequent decline in CLABSI rates. The ITS regression equation model was Y (CLABSI Rate) = 6.069 − 0.021 (T) + 5.791 (Xt)- 0.947 (TXt).

**Table 3:** Interrupted Time Series Analysis Coefficients

	Estimate	Std Error	T-value	p-value	2.5%	97.5%
(Intercept)	6.069	3.418	1.776	0.091	-1.061	13.198
Time	-0.021	0.464	-0.044	0.965	-0.989	0.948
Intervention	5.791	4.558	1.271	0.218	-3.717	15.299
Time after Intervention	-0.947	0.657	-1.442	0.165	-2.317	0.423

**Fig. 3:**
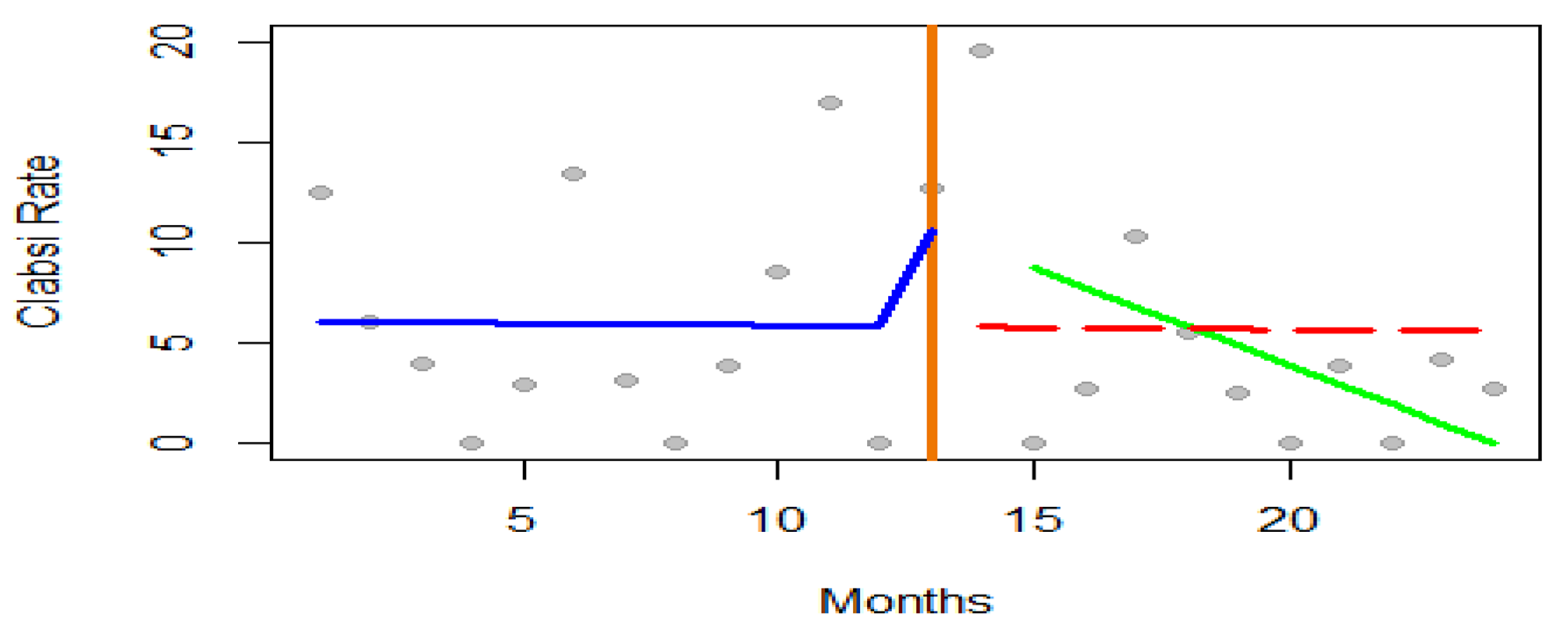
Trend line of monthly CLABSI Rate before and after intervention

The trend graphically is represented in Fig. 3; the Blue line- shows the Trend during pre-intervention. Redline- Predicted trend without intervention. Green line- Trend after the intervention. Orange line – Project Intervention start period, grey dots- CLABSI rates per 1000 catheter days.

## Discussion

This study highlights the importance of utilizing theoretical models, such as the “ Plan, Do, Study and Act”, to enhance transparency and reproducibility in quality improvement projects [17, 25]. Clear reporting of methods, interventions, and outcomes can facilitate knowledge sharing and allow other healthcare facilities to learn from both successful and unsuccessful experiences [16]. During the intervention period, there was an immediate increase in the CLABSI rate, which then started to decrease as the intervention was ongoing. Although a decline of 18% during the intervention was reported, this was not strong enough to show statistical significance [26, 27].

The lack of statistical significance in this study may also indicate that other factors beyond the implemented interventions may have significantly influenced the occurrence of CLABSI [11]. Another factor that may have had a confounding effect on this outcome is the decline in COVID-19 cases during the intervention period as opposed to during pre-intervention period, when COVID-19 cases were very high in our critical care areas., However, the impact of COVID-19 on CLABSI rates was not accessed in our analyzed results [28]. The potential impact of the COVID-19 pandemic on increased CLABSI rates during the pre-intervention period may have been specifically because of practices around PPE requirements for healthcare providers and inappropriate use of antibiotics which may have played a role in the increased CLABSI rates [29]. It is crucial to consider the complex and multifactorial nature of CLABSI and acknowledge that multiple factors, including patient characteristics, healthcare practices, and environmental factors, can contribute to infection rates.

This study contributes to the body of knowledge on CLABSI prevention by highlighting the challenges and complexities associated with implementing interventions in real-world healthcare settings. It underscores the need for continuous monitoring, evaluation, and adaptation of interventions to achieve sustainable improvements in patient outcomes, the need to determine the actual adherence of each item in the bundle and investigate reasons for non-adherence [6]. Future studies could explore additional interventions or strategies to address other contributing factors.

### Limitations

The lack of secondary validation of reported compliance rates by assigned unit educators on adherence by direct observation for central line care bundles may have overestimated compliance and limited the project’s ability to establish a causal relationship between the interventions and CLABSI reduction. Data collection was done manually, increasing the risk of incomplete data, especially during nights and weekends, and labor intensive which would have potentially affected the accuracy of the bundles compliance rates. .

## Conclusions

This study provides insights into the challenges and outcomes of quality improvement projects that are aimed at reducing CLABSI rates in healthcare facilities. It points out the need for alternative approaches and strategies to effectively prevent CLABSI and improve patient outcomes. Although there was an observed decline of 18% in CLABSI rates during the intervention period, this decline did not reach statistical significance suggesting that improved compliance to central line care bundles alone may not be strong enough to significantly reduce CLABSI rates and that other factors unrelated to bundles compliance may have also influenced CLABSI occurrence.

This study also conceptualize the importance of using theoretical models like the “Plan, Do, Study, and Act” framework in quality improvement projects to enhance transparency and reproducibility. Clear reporting of project methods, interventions, and outcomes is crucial for knowledge sharing and learning for both successful and unsuccessful experiences. And, there is need for continuous monitoring, evaluation, and adaptating interventions to achieve sustainable improvements in patient outcomes. Observed practice may influence compliance towards the desired standards.

Future research should explore additional interventions or strategies as a comprehensive approach to infection prevention that can address other contributing factors to CLABSI.

## Data Availability

The data that were generated and analyzed for this study are included in this manuscript.

## Abbreviations

CLABSI: Central line-associated bloodstream infection
CQI: Continuous quality improvement
CVC: Central Venous Catheter
DOT: Days of Therapy
HAI: Hospital Associated Infection
ID: Infectious Disease
ITS: Interrupted Time Series
IPC: Infection Prevention and Control
NHSN: National Health and Safety Network
SQUIRE: Standards for Quality Improvement Reporting Excellence
